# Supplementary material for: Field evaluation of thermal and acoustical comfort in eight North-American buildings using embedded radiant systems
Source: PLoS One. 2021 Oct 26;16(10):e0258888. doi: 10.1371/journal.pone.0258888 (PMC8547689; doi:10.1371/journal.pone.0258888)
Supplement: Attachment — Submitted filename: Review v1 Dawe et al. PLOS One.pdf [file pone.0258888.s002.pdf]

Below are my comments on your manuscript. I have organized the review into (A) high level comments that I believe will help the overall character of the paper but are at the discretion of the authors as to whether they are worth implementing and (B) detailed comments that I believe should be addressed.

(A) High level comments

Overall, this text is extremely clearly written, and I am left with few questions about the study that was conducted.

1. Is it possible to add floor plans or photographs of each building/space analyzed. I agree with your comment in the paper that a drawback of the 8 building sample size is the lack of generalizability. Therefore, as a reader, I would be very interested in attempting to draw my own conclusions about the data for each case, something that can be done with such a small sample size. This is a unique quality to your paper. It would be beneficial to provide as much context to the study as possible. Particularly in conjunction with tale 2, it would be very helpful to be able to understand whether a building with a trickle vent also has an open plan. Annotations about where individuals who participated in the comfort study were located within a larger floor plan would be helpful. In general, as much supporting detail for such a small sample size would be very interesting and help provide more insight for such a small sample size. Even if this level of detail is not allowed or possible, photos of sites or systems would be helpful. Clearly building 2 has anonymity concerns, but in a paper with so few cases the narrative would be greatly enhanced with more direct engagement with the sites.
2. Your limitations section should be expanded into a general discussion section. One thing that comes to mind to add here is a comparison to the companion paper mentioned in the introduction, but seemingly not again, about how your findings align with the previous work. Further, your objectives are to help inform designers and building operators address comfort and wellbeing issues in radiant buildings. Your results section is very matter-of-fact, and then you list limitations. So either the scope of the analysis was too small to achieve this objective, which should be stated, or you should directly list these recommendations and more casually discuss the results in an expanded discussion section. One idea would be for an interpretation of the results in the context of floor plans. Another would be to compare your results to previous work. Another is to subjectively comment on the huge EUI range and perhaps normalize this number by occupant density and think about whether this new normalized metric correlates better with your data. And would also explain if the number of respondents per building, n, is proportional to the population or not.
3. In general, I think the paper would benefit if the responses were broken down to heating versus cooling cases. I think issue 3 in section B below would be clarified quite substantially if the results were split into heating or cooling mode. A lot of text in the paper is dedicated to describing conformity to ASHRAE satisfaction standards. To some extent this is less exciting and you may consider moving these results to the supplementary material, unless they are greatly different from all air buildings or other results accumulated from the 26 other radiant buildings. What may be interesting to explore further is cross-correlations between thermal comfort responses, such as is a

person that is too hot and asked for more air motion less likely to be susceptible to drafts in radiant heating modes? These types of data would be very novel and very helpful to understand how occupants respond to radiant environments. It would also help assess whether radiant heat is confusing to occupants and issues are improperly associated with air conditions. I think this type of autocorrelation data would make this an exceptional paper. I ask this because the data in figure 2A is well presented, but it begs to be further engaged with.

(B) Detailed comments

1. Why were buildings with TABs or ESS systems selected? Please add your criteria rationale to the objectives section (3).
2. Can you provide more detail about ESS systems? I may be thinking about this incorrectly, but do the ESS systems in the studied spaces have faster or slower response times than TABs? It would be good to quantify the time constants of the slab response time and ESS response time. Clarification about the difference between TAB and ESS would be helpful for a broad audience. Metallic hydronic radiant panels are fairly common and would certainly have much faster response times. It may be worth mentioning these in response to the slow system response time as a future design consideration.
3. Line 300 - This is a really interesting point that is worth diving into deeper. First, was this comment about air speed to low during cooling or heating? My guess is cooling, but this should be clarified. Radiant systems benefit in terms of energy performance by allowing for air temperature setbacks, and in cooling mode this means that as air gets warmer it is approaching skin temperature. When this happens, less heat is removed from the skin at the same air speed. In fact, one can calculate that there is an exponential dependence in the air speed for equivalent heat transfer as air temperature approaches skin temperature. Instead of being a limiting factor for radiant systems, this finding is very interesting in rethinking these comfort physics which could help inform future radiant system design in conjunction with elevated air speed. This explanation could be helpful, especially in the context of cooling or heating mode. That said, figure 2 shows that air movement too low and area colder than others are the 3rd and 4th most prevalent thermal discomfort reasons. Are these from the same surveys, and therefore in heating or cooling mode? This is a very interesting finding if people want more air motion in heating mode or are too cold in cooling mode.
4. Section beginning at Line 310 - I think that it gives building occupants a lot of credit to attribute thermal comfort issues to surfaces. Were participants prepped in any way or given any kind of education on the building systems? I think the statistics in this paragraph are fairly meaningless if not. The paragraph then goes on to identify the buildings as designed with direct solar heat gains avoided, which is successful at minimizing direct shortwave exposure. This is different from the results of hot or cold walls being discussed in the previous sentences, and should be moved to the methods describing the buildings. In addition, reference 24 was provided as a source for not having external walls in the view

factor of occupants. This is a very important point, and something that floor plans would greatly help with. At the very least this should be mentioned earlier or added as a column in a table whether external walls are in the occupied space. Still, ~15% of occupants cite incoming sun as a reason for discomfort in figure 2A. This seems to not match the narrative.

5. Paragraph beginning at line 319 - please describe also whether uniformity is in reference to air temperature, radiant temperature, or both.
6. Is the EUI a post-occupancy measured EUI or a design EUI? The enormous discrepancy should be discussed more, and also perhaps an EUI per person should be calculated. You mention that the air side systems may be responsible for overcooling and therefore the relatively large EUI, but these values are also enormous for all air buildings, no? Curious if it is perhaps an occupant density issue as well. Requiring an additional system to treat latent loads usually is for locations with very large internal latent loads, pointing t high occupant density.

I hope that these comments are interesting and provide some exciting sources of academic debate during the response process. I am very excited about radiant technology, and think that you have very exciting findings embedded in your data. I look forward to continue the conversation!

Eric Teitelbaum
