# Supplementary material for: Field evaluation of thermal and acoustical comfort in eight North-American buildings using embedded radiant systems
Source: PLoS One. 2021 Oct 26;16(10):e0258888. doi: 10.1371/journal.pone.0258888 (PMC8547689; doi:10.1371/journal.pone.0258888)
Supplement: Attachment — Submitted filename: Response to Reviewers.docx [file pone.0258888.s003.docx]

**Reviewer 1 High-Level Comments:**

Is it possible to add floor plans or photographs of each building/space analyzed. I agree with your comment in the paper that a drawback of the 8 building sample size is the lack

of generalizability. Therefore, as a reader, I would be very interested in attempting to

draw my own conclusions about the data for each case, something that can be done

with such a small sample size. This is a unique quality to your paper. It would be

beneficial to provide as much context to the study as possible. Particularly in conjunction

with tale 2, it would be very helpful to be able to understand whether a building with a

trickle vent also has an open plan. Annotations about where individuals who participated

in the comfort study were located within a larger floor plan would be helpful. In general,

as much supporting detail for such a small sample size would be very interesting and

help provide more insight for such a small sample size. Even if this level of detail is not

allowed or possible, photos of sites or systems would be helpful. Clearly building 2 has

anonymity concerns, but in a paper with so few cases the narrative would be greatly

enhanced with more direct engagement with the sites.

Response:

We agree that more context and details about the buildings will be useful. Our post-occupancy evaluation (POE) approach was to find shared thermal comfort themes across several buildings rather than to focus on the specific and unique outcomes of each individual building. We acknowledge that different buildings can result in different occupant experiences (which is addressed in section 3.4.4 of Karmann et al. 2017). Where unique operation or feedback in a building seemed meaningful to us, we highlighted those instances in the “Miscellaneous sources” of Section 5.2.2, and called out the limitation to generalize the finding across all buildings. We do not have our own photos or floor plans to include. We agree that this could help readers understand the nuances at each building and wish we had them. We are also concerned that adding generic visuals (instead of our own) would not necessarily support the arguments of this article and these would primarily serve as a decorative purpose, which would not be relevant. POE studies come in very different levels of details, from the simple and generic surveys, like the CBE Occupant Survey to very detailed case studies that includes focused group, structured interview, physical measurements of energy, water, thermal comfort, acoustics, lighting, air quality, and cost analysis. One example of a detailed study is described in <https://escholarship.org/uc/item/30h937bh>. The study presented here falls in the range between these two extremes. We have general information about occupants’ location in the buildings, such as floor number and orientation. We investigated those for trends, but we did not find anything generalizable or meaningful across the building populations. We did not obtain specific information on individuals’ locations to maintain anonymity for occupants that is a requirement of the IRB.

Your limitations section should be expanded into a general discussion section. One thing

that comes to mind to add here is a comparison to the companion paper mentioned in

the introduction, but seemingly not again, about how your findings align with the previous

work. Further, your objectives are to help inform designers and building operators

address comfort and wellbeing issues in radiant buildings. Your results section is very

matter-of-fact, and then you list limitations. So either the scope of the analysis was too

small to achieve this objective, which should be stated, or you should directly list these

recommendations and more casually discuss the results in an expanded discussion

section. One idea would be for an interpretation of the results in the context of floor

plans. Another would be to compare your results to previous work. Another is to

subjectively comment on the huge EUI range and perhaps normalize this number by

occupant density and think about whether this new normalized metric correlates better

with your data. And would also explain if the number of respondents per building, n, is

proportional to the population or not

Response:

We have retitled Section 5 to Results and Discussion. We do not feel that a standalone discussion section is necessary due to the qualitative nature of the study. To develop the Results, we parsed through and assembled hundreds of individual occupant responses and open-ended feedback into common themes, which are communicated in Section 5. We draw on occupant feedback to provide designers and building operators with information on how occupants experience the space and suggestions to improve thermal comfort. We have expanded and strengthened the directness of these suggestions in the section.

In response to your suggestion to compare our results to previous work, we tried to do it in the discussion of **thermal comfort uniformity** in Section 5.2.2, as this was one of the most commonly cited thermal comfort feature in literature. We have strengthened this discussion.

In response to your comment on the large EUI range and suggestion to normalize by building occupants: the wide EUI range was obtained by design. We intentionally selected the buildings with large variation in energy and comfort from a large pool analyzed in Karmann et al.’s paper (as stated in section 4.1). We selected buildings with varying EUI to investigate the relationship between energy efficiency and thermal comfort. We have added this in 4.1 Building selection, as well as clarified EUI includes all sources of energy. In addition to the EUIs reported in the study, we have added the EPA EnergyStar Score to Table 1, which is normalizes energy use according to building size, location (climate), number of occupants, operating hours, and number of computers. among other factors for commercial buildings. More information on ENERGY STAR score can be found here <https://www.energystar.gov/buildings/tools-and-resources/energy-star-score-offices>

In general, I think the paper would benefit if the responses were broken down to heating

versus cooling cases. I think issue 3 in section B below would be clarified quite

substantially if the results were split into heating or cooling mode. A lot of text in the

paper is dedicated to describing conformity to ASHRAE satisfaction standards. To some

extent this is less exciting and you may consider moving these results to the

supplementary material, unless they are greatly different from all air buildings or other

results accumulated from the 26 other radiant buildings. What may be interesting to

explore further is cross-correlations between thermal comfort responses, such as is a

person that is too hot and asked for more air motion less likely to be susceptible to drafts

in radiant heating modes? These types of data would be very novel and very helpful to

understand how occupants respond to radiant environments. It would also help assess

whether radiant heat is confusing to occupants and issues are improperly associated

with air conditions. I think this type of autocorrelation data would make this an

exceptional paper. I ask this because the data in figure 2A is well presented, but it begs

to be further engaged with.

Response:

In previously published work (Karmann et al. 2017) we showed that only a tiny percentage of buildings meet ASHRAE 55-2017 objective of achieving 80% satisfaction rate. Given ASHRAE 55’s importance in guiding the design and operation in the US and often globally, we feel that this section is relevant and plan to keep it in its current location within the paper. In response to the comment, we have added the high-level comparison values between the entire radiant and all-air datasets from the companion paper and shortened the section by removing one of the thermal comfort definitions which does not come from ASHRAE 55. We have done the same in the matching Acoustics section (5.3.1)

You suggested breaking responses out into heating or cooling mode. Occupant surveys are intended to gather occupant’s long-term experience (e.g., over the past 6-months), which is stated to them at the onset of the survey. We did not target a particular operation mode. Additionally, the occupant’s thermal sensation (e.g., hot, cold) would be needed in addition to operation mode of the system to make the suggested analysis more meaningful. We agree with your suggestions and feel these would be covered with a “right-now” survey.

We acknowledge your suggestions to examine cross-correlations between individual’s responses regarding thermal comfort. We agree this could be informative but feel that digging into further layers to seek statistical significance will not be fruitful due to the sample size in the study. This was attempted in part by Karmann et al. 2017 (section 3.3.4) on the full 26 radiant buildings. The authors did not find any meaningful outcomes of the exercise. A larger dataset is needed to provide something reliable. Right now, we are working on this for the entire CBE Occupant Survey database (~90,000 persons), a large sample is needed to find meaningful relationship.

**Reviewer 1 Detailed Comments:**

Why were buildings with TABs or ESS systems selected? Please add your

criteria rationale to the objectives section (3)

Response:

We selected these building systems because we expect they would have higher energy savings, lower cost, and the possibility of thermal storage (grid to building load flexibility). Moreover, embedded systems have a much longer time constant than panel systems, and may have thermal comfort and acoustical issues, so their concern were specific. We have added this to 4.1 Building selection.

Can you provide more detail about ESS systems? I may be thinking about this incorrectly, but do the ESS systems in the studied spaces have faster or slower response times than TABs? It would be good to quantify the time constants of the slab response time and ESS response time. Clarification about the difference between TAB and ESS would be helpful for a broad audience. Metallic hydronic radiant panels are fairly common and would certainly have much faster response times. It may be worth mentioning these in response to the slow system response time as a future design consideration

Response:

ESS has a faster response time than TABS most of the time, but both are generally slower than panels.

We have already provided a reference to the study from Baisong Ning (see figure 6 in ref 23) which the response times of thousands of different radiant systems were calculated. We have specifically added more detail to section 4.1 Building Section and Section 5.2 under “ability to quickly and individually…” so it is clear for future readers.

Line 300 - This is a really interesting point that is worth diving into deeper. First,

was this comment about air speed to low during cooling or heating? My guess is

cooling, but this should be clarified. Radiant systems benefit in terms of energy

performance by allowing for air temperature setbacks, and in cooling mode this

means that as air gets warmer it is approaching skin temperature. When this

happens, less heat is removed from the skin at the same air speed. In fact, one

can calculate that there is an exponential dependence in the air speed for

equivalent heat transfer as air temperature approaches skin temperature. Instead

of being a limiting factor for radiant systems, this finding is very interesting in

rethinking these comfort physics which could help inform future radiant system

design in conjunction with elevated air speed. This explanation could be helpful,

especially in the context of cooling or heating mode. That said, figure 2 shows

that air movement too low and area colder than others are the 3rd and 4th most

prevalent thermal discomfort reasons. Are these from the same surveys, and

therefore in heating or cooling mode? This is a very interesting finding if people

want more air motion in heating mode or are too cold in cooling mode.

Response:

In general, we agree with your observations and comments. However, the survey is a long-term survey meant to provide general feedback from occupants’ experience in the space rather than instantaneous feedback. We cannot report back on further details that the reviewer requests because we do not have information on the temperature in the space or operation mode when occupants experience these sensations. This type of analysis can be done with right-now surveys.

We have cleared up the language because realize it reads as though we have “right now” survey responses. We can only speculate about system operation and occupant thermal sensation.

You may find interesting the chart in the CBE Thermal Comfort Tool named “Thermal Heat Losses vs Air Temperature” and “SET output”, they are two of the five charts that can be visualized on the right. There, you can explore how latent and sensible heat losses changes as faction of air temperature when using the PMV or SET models.

Section beginning at Line 310 - I think that it gives building occupants a lot of

credit to attribute thermal comfort issues to surfaces. Were participants prepped

in any way or given any kind of education on the building systems? I think the

statistics in this paragraph are fairly meaningless if not. The paragraph then goes

on to identify the buildings as designed with direct solar heat gains avoided,

which is successful at minimizing direct shortwave exposure. This is different

from the results of hot or cold walls being discussed in the previous sentences,

and should be moved to the methods describing the buildings. In addition,

reference 24 was provided as a source for not having external walls in the view

factor of occupants. This is a very important point, and something that floor plans

would greatly help with. At the very least this should be mentioned earlier or

added as a column in a table whether external walls are in the occupied space.

Still, ~15% of occupants cite incoming sun as a reason for discomfort in figure

2A. This seems to not match the narrative.

Response:

We generally agree that this section could be improved and the noted discrepancy between shortwave direct solar and longwave hot/cold surfaces. Based on comments, many occupants understood that they are in a building with a radiant system, but we tend to agree that this does not prove that an occupant understands the heating or cooling mechanisms of the system. The fact that occupants did not report surfaces as a source of discomfort could be attributed to lack of knowledge. We have revised this section accordingly to remove any conclusions or suggestions.

In regards to reference 24 and discussion on viewable surfaces: we have removed this because we removed any suggestions related to hot/cold surfaces and this reference may cause confusion. Our study did not investigate whether, specifically, external walls were the cause of discomfort. Our intent with this reference was to speculate why occupants were not identifying hot or cold surfaces as sources of discomfort. One theory shown in ref 24 is that in a space with an active radiant surface, all surfaces that are within view of the active surface are exchanging heat with the active radiant surface, and thus, reducing temperature difference between surfaces, including external walls (ref 24). Therefore, potentially avoiding any one surface or external wall from causing discomfort.

Paragraph beginning at line 319 - please describe also whether uniformity is in

reference to air temperature, radiant temperature, or both.

Response:

This is an excellent question and we have slightly expanded our discussion here. Our literature review identified common thermal comfort findings in radiant buildings. We came across the terms “temperature uniformity” or “thermal comfort uniformity” in several references, including the REVHA Guidebook. However, the terms appear to have different definitions across the literature and is not well-defined in general. That leaves a lot of ambiguity. We have revised the section accordingly, and chosen the term “thermal comfort uniformity” for our assessment. We assess thermal comfort uniformity based on subjective occupant responses, because we did not have objective temperature measurements in the space to evaluate temperature uniformity or PMV. We have clarified our terminology and limitation to assess temperature uniformity.

Is the EUI a post-occupancy measured EUI or a design EUI? The enormous

discrepancy should be discussed more, and also perhaps an EUI per person

should be calculated. You mention that the air side systems may be responsible

for overcooling and therefore the relatively large EUI, but these values are also

enormous for all air buildings, no? Curious if it is perhaps an occupant density

issue as well. Requiring an additional system to treat latent loads usually is for

locations with very large internal latent loads, pointing t high occupant density.

Response:

EUI is post-occupancy based on metered utility data. As we have described in an earlier response, the discrepancy was designed, not perchance. We do not have enough system-specific energy consumption information to speculate as to the cause of higher EUIs, and have noted that the EUIs include all energy sources. These values were reported as kBtu/ft2 in the referenced paper, converted to kWh/m2 for SI units. We have included the EPA ENERGY STAR Scores in Section 5.1, which normalize for building characteristics, as described above.

**Reviewer 2 Comments:**

In section 4.1, you should add a caveat to the use of TABS.

Compared to radiant panels, TABS has a slow response time while panels are the fastest responding system. This is a significant difference. Another critical difference is that TABS has challenging control and basically, setpoints don't exist. The reason is that temperature measured across the slab thickness does not reflect slab surface temperature, especially when covered with carpet.

Response:

We agree and have already included reference to a paper that addresses response time of various radiant systems (ref. 23: Baisong Ning et al.). Additionally, we have added language around response times for TABS and ESS to Section 4.1 and 5.1. We focused on a subset of radiant systems with high thermal mass (TABS and ESS), and do not wish to bring in the discussion of radiant panels to avoid confusing our audience.

In section 4 or section 5 or the Limitation section, you need to recognize the limitation of the data collection method. This is a widely used data collection method, but it has an inherent error that results can be biased, especially when we have low response rates. Lassen et al. (2020) reported this phenomenon. Discussion of this issue required a separate paper, so I am only asking to recognize this limitation, hoping that the scientific community will improve data collection or better understand the results. For example, we will understand what it means to have 30 responses out of 300 occupants, who is responding or voting and why. You address other issues very well in section 5.

Lassen, N., Goia, F., Schiavon, S. and Pantelic, J., 2020. Field investigations of a smiley-face polling station for recording occupant satisfaction with indoor climate. Building and Environment, 185, p.107266.

Response:

We agree response rate is an important consideration for any survey method, especially when it is low, and will add this as a limitation. We acknowledge that non-response bias should be highlighted and the higher the response rate, the more representative the result.

In Section 4.3 please add categories used. For example, how did you categorize information you received about the radiant system design?

Response:

We have added additional information in section 4.3 with 2-3 questions that were part of the radiant characteristics survey. Additionally, the information in Table 2 come from the survey.

Please also include some indication of heating and cooling operation. How many buildings used both vs only one of these options.

Response:

This item is brought up by both reviewers. We have added that all buildings use both radiant heating and cooling to Section 5.1. The original dataset required radiant cooling, and per chance, all of these buildings also use radiant heating.

Occupant surveys are intended to gather information on long-term experiences in the building. We cannot reliably comment on whether occupant responses are in reference to heating or cooling mode of the system because we did not collect that information. We have added indication of whether solutions tend to be more applicable when occupant’s feel warm or cool (regardless of system operation).

Line 276 - response time of the TABS is the essential factor. Another factor is the temperature of the surface that is exchanging heat with the occupants. How do we modulate that temperature? Slab temperature, slab surface temperature, and carpet (or similar top covering of the slab exchanging heat with the occupant) can have significantly different temperatures. You can find more details in Pantelic et al., (2018). Please discuss this issue also.

Pantelic, J., Schiavon, S., Ning, B., Burdakis, E., Raftery, P. and Bauman, F., 2018. Full scale laboratory experiment on the cooling capacity of a radiant floor system. Energy and Buildings, 170, pp.134-144.

Response:

We agree and do not feel that line 276 suggests otherwise. Your comment suggests that we include discussion of temperature control method as it relates to thermal comfort. In our initial investigation, we looked at radiant system control (constant/variable temperature and constant/variable flow) and temperature setpoint control methods (air temperature setpoint, slab temperature setpoint, etc.), as you suggest. In this small sample, we did not find strong correlation between system control and thermal comfort. System and temperature control methods varied, and there was simply not a large enough sample size to have a suitable comparison.

Line 350 - how many design strategies you evaluated? In Table 2 there is a summary of the systems, but DOAS means that airside supplies only outdoor air, and there is no information about the size of air vs. radiant system. Radiant loop control would be equivalent to coil water side control that we never really consider in any discussion on thermal comfort, so I think this is a wrong parallel. As I mentioned above, the critical problem besides slow response is what is setpoint controlling? Perhaps this is a good place to discuss this since you mentioned other design issues.

Response:

We did not select buildings based on specific radiant design or control strategies; therefore, no two designs were exactly the same. This is an inherent challenge with field studies. We are able to group similar design and control strategies (seen in Table 1 of Section 5.1) to evaluate thermal comfort.

To be included in the study, radiant had to be the primary cooling mechanism for the space. We have also included indication of supplemental cooling systems in Table 2 of Section 5.1.

We understand your comments and feel the correct response is to clarify the intent of Line 350 (now line 387) and onward. Based on the sample of buildings in this study, there was no strong evidence correlating thermal comfort and building design or radiant system control, with the exception of one scenario where building operators were trying to control the radiant zones like a forced air system by changing setpoints. This suggests that design and operation may not have significant impacts on comfort – as long as the method of operation is appropriate for radiant systems. We edited this paragraph to include the various control strategies available and highlight that this is not a large enough sample size to have a definitive observation.

Lines 375-380 - this is interesting information. I understand that you can't make a significant conclusion based on the information provided, but it certainly points out an issue that needs further investigation.

Response:

We agree . We have added this to the Miscellaneous sources in 5.2.2.

In the Conclusion section, you mention that one of the comfort improvement methods is to use a personal fan. That is true for warm conditions in summer. What would be the method for winter operation and cold conditions?

Response:

Some occupants had personal heaters, we have added this to the conclusion section and it is already called out in 5.2.2.
